# Discrimination of benign and malignant ovarian sex cord-stromal tumors through the analysis of clinical features, MR imaging, and MR-based radiomics

**DOI:** 10.3389/fonc.2026.1817093

**Published:** 2026-05-25

**Authors:** Shifang Tan, Lingjie Zhang, Zhexuan Yang, Xueyan Liu, Meiying Cheng, Mingsheng Yi, Honglei Shang, Zhanqi Feng, Jingxu Xu, Chencui Huang, Xiaoan Zhang, Xin Zhao

**Affiliations:** 1 Department of Radiology, The Third Affiliated Hospital of Zhengzhou University, Zhengzhou, China; 2 Department of Research Collaboration, R&D center, Hangzhou Deepwise & League of PHD Technology Co., Ltd, Hangzhou, China; 3 Tianjian Laboratory of Advanced Biomedical Sciences, Institute of Advanced Biomedical Sciences, Zhengzhou University, Zhengzhou, China

**Keywords:** diagnostic performance, machine learning, magnetic resonance imaging, radiomics, sex cord-stromal tumor

## Abstract

**Objectives:**

This study aims to compare diagnostic performance of clinical parameters, conventional MRI, and radiomic signatures, and to develop a multimodal nomogram for differentiating benign from malignant ovarian sex cord-stromal tumors (SCSTs).

**Methods:**

Retrospectively enrolled 113 patients with pathologically confirmed 123 SCSTs (42 malignant and 81 benign). Univariate and multivariate logistic regression analysis identified the independent predictors for clinical and conventional MRI models. Radiomics features were extracted from fat-suppressed T2-weighted imaging (FS-T2WI) and diffusion-weighted imaging (DWI) sequences, with extreme gradient boosting (XGBoost) used to build radiomics models. A combined nomogram integrated Rad-scores with the independent clinical and conventional MRI predictors. Model performance was evaluated by receiver operating characteristic (ROC) analysis and decision curve analysis (DCA) in development and validation cohorts.

**Results:**

Among traditional methods, the traditional model achieved the highest AUCs (training: 0.880 [*95% CI*: 0.807-0.946]; validation: 0.908 [*95% CI*: 0.797-0.989]). Among radiomics-based models, T2&DWI model achieved the highest AUCs (training: 0.887 [*95% CI*: 0.802-0.955]; validation: 0.843 [*95% CI*: 0.684-0.963]). The combined model demonstrated optimal predictive performance across all models, with AUCs of 0.945 (*95% CI*: 0.892-0.985) in training and 0.914 (*95% CI*: 0.798-0.988) in validation cohorts, respectively. Furthermore, DCA confirmed greater clinical net benefit of the combined model across threshold probabilities compared to other models.

**Conclusion:**

While both traditional model and MRI-based radiomics model demonstrate significant potential for SCSTs stratification, our final combined nomogram achieves optimal predictive performance and net benefit. This tool may enhance personalized therapeutic management for SCSTs patients.

## Introduction

Sex cord-stromal tumors (SCSTs) constitute a rare category of ovarian neoplasms, accounting for approximately 7-8% of all ovarian tumors (1, 2). This histologically heterogeneous group encompasses both benign and malignant entities with distinct clinicopathological characteristics and biological behaviors (3). The most prevalent malignant subtype is granulosa cell tumor (GCT), representing the majority of malignant SCSTs, while fibromas dominate the benign spectrum as the most frequently encountered subtype (1, 4). Other subtypes of SCSTs (including thecomas, sclerosing stromal tumors, Sertoli Leydig cell tumors, etc.) are relatively rare.

The diagnostic evaluation of SCSTs presents significant challenges due to three interrelated factors: the remarkable histopathological heterogeneity across subtypes, their overall rarity (that disproportionate to their variety), and frequent overlap observed both within the SCST subcategories and with non-SCST ovarian neoplasms (3, 5). Given the different biological behaviors and prognoses between benign and malignant tumors, an accurate preoperative identification is particularly critical for guiding therapeutic and management strategies, and this clinical imperative is compounded by the demographic distribution of SCSTs, which tend to affect younger women of reproductive age. In this patient population, conservative surgery approaches to preserve optimal reproductive and endocrine function are prioritized when oncologically feasible (1, 3, 6).

Owing to the superior soft tissue contrast resolution, Magnetic resonance imaging (MRI) has been widely used to detect and evaluate adnexal lesions, especially for the indeterminate adnexal masses on ultrasonography (US) (7, 8). Previous studies have preliminarily demonstrated the diagnostic potential of MRI in distinguishing benign from malignant SCSTs (9, 10), nevertheless the inherent subjectivity in image interpretation, compounded by the aforementioned diagnostic challenges specific to SCSTs (including their morphological heterogeneity and rarity) significantly exacerbates the research complexity. These underscore substantial research potential in imaging-based differentiation strategies for SCSTs, and driving the demand for further investigation into advanced imaging biomarkers to optimize SCSTs management.

Radiomics utilizes cutting-edge artificial intelligence (AI) algorithms to transform imaging data into quantitative features, which can be subsequently examined with the aim of enhancing diagnostic and prognostic accuracy while providing personalized treatment options for patients, in recent years, radiomics provide a diverse array of potential applications in the field of oncology (11). Despite these advances, radiomics in gynecologic oncology does not meet the threshold for daily clinical application compared with other medical fields (12), with particularly limited exploration in SCST subtyping. Existing studies on SCST imaging are hampered by small sample sizes, lack of validation, and restricted sequence usage, highlighting the need for more robust studies. In this study, we aimed to develop and compare multiple predictive models based on clinical parameters, conventional MRI characteristics, and radiomic signatures for discriminating benign from malignant SCSTs. Specifically, we sought to identify the optimal model with the highest diagnostic performance, which could serve as a preoperative stratification tool to assist clinical decision-making.

## Materials and methods

### Patient sample

The institutional review board of the Third Affiliated Hospital of Zhengzhou University approved this retrospective study (2023-252-01) and granted a waiver of informed consent due to the utilization of anonymized data. Our study conformed to the Declaration of Helsinki on Human Research Ethics standards.

**Figure 1:**
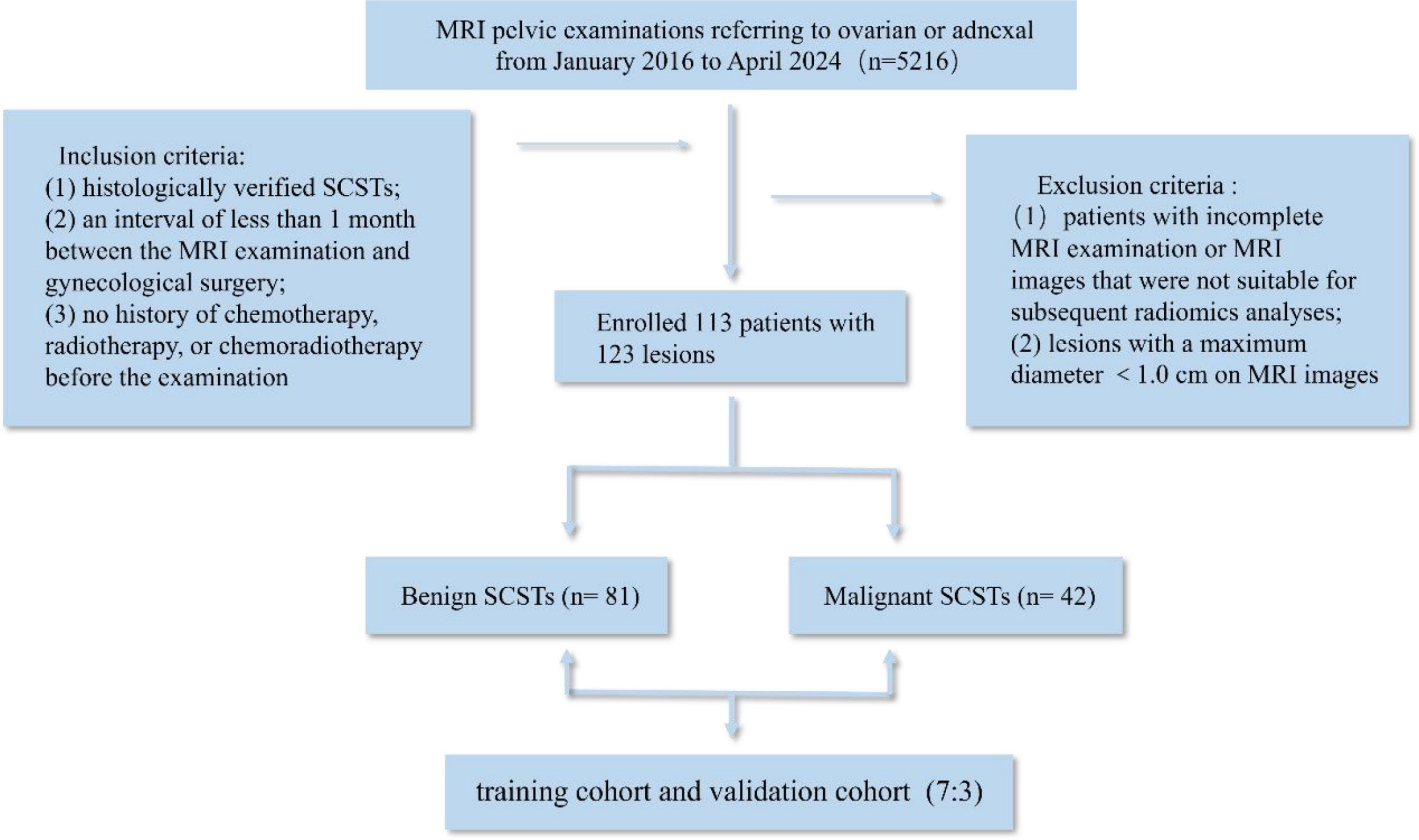
Flowchart of the patient cohort.

All patients who underwent pelvic MRI examinations referring to ovarian or adnexal lesions between January 2016 and April 2024 were evaluated. This evaluation task was carried out by two radiologists (Liu XY and Yi MS, with 3 and 9 years of experience, respectively). The inclusion criteria were: (1) histologically verified SCSTs; (2) an interval of less than 1 month between the MRI examination and gynecological surgery; (3) no history of chemotherapy, radiotherapy, or chemoradiotherapy before the examination; The exclusion criteria were: (1) patients with incomplete MRI examination or MRI images that were not suitable for subsequent radiomics analyses; (2) lesions with a maximum diameter <1.0 cm on MRI images. A flowchart of the patient selection process is summarized in **Figure 1**.

### Clinical data

The clinical information was collected through Hospital Information System (HIS) and recorded by two physicians (Yang ZX and Feng ZQ, both with 4 years of experience). Clinical variables encompassed (1) Age: continuous variable; (2) Menstrual status: categorized as “premenopausal” or “postmenopausal”; (3) Clinical symptoms: categorized as “asymptomatic”, “menstrual disorders”, “frequent micturition”, “abdominal pain”, or “palpable lump”; (4) Hormone levels: categorized as “normal” (within the laboratory reference interval (RI)), “mild elevation” (1.0 – 2.0 × upper RI), or “significant elevation” (> 2.0 × upper RI); (5) Carbohydrate antigen 125 (CA125) levels: categorized as “normal” (< 35 μg/L), “mild elevation” (35 – 200 μg/L), or “significant elevation” (> 200 μg/L); (6) Pelvic effusion: including isolated pelvic effusion or effusion accompanying ascites, categorized as “none”, “mild”, or “severe” by depth.

### MRI acquisition protocol

MRI examinations were performed on the 3T MR scanner (SIGNA Pioneer, GE Healthcare, and Skyra, Siemens Healthcare) with the 18-channel abdominal phased array coil. The imaging protocol included axial and sagittal T2-weighted imaging (T2WI) without fat saturation, axial and coronal T2-weighted imaging with fat saturation (FS-T2WI), axial T1-weighted (T1WI) in- and opposed-phase images, axial diffusion-weighted imaging (DWI), and three-dimensional dynamic contrast enhanced MRI (DCE-MRI), which is a dynamic FS-T1WI sequence performed before and after intravenous contrast material administration. Detailed information about the acquisition parameters is presented in supplementary materials (**Supplementary Table 1**).

### Conventional MRI imaging feature

MRI images were obtained through Picture Archiving and Communication System (PACS). Two radiologists (Zhang LJ and Tan SF, with 5 and 9 years of experience in MRI, respectively) who were blinded to the clinic-pathologic data independently evaluated and recorded the conventional imaging features, including (1) Shape: categorized as “regular” (approximate rounded/elliptical contours) or “irregular” (spiculated/lobulated contours); (2) Margin: categorized as “sharp” or “indistinct”; (3) Maximum diameter (MD): continuous variable, the largest diameter of the lesion in three-dimensional measurement; (4) Lesion types: categorized as “predominantly solid” (lesions composed of more than 2/3 enhancing solid tissue), “cystic-solid” (lesions composed of 1/3-2/3 solid tissue), or “predominantly cystic” (lesions composed of less than 1/3 solid tissue); (5) Peripheral cystic areas: categorized as “absent” or “present”; (6) Signal intensity of the solid components on T2WI (T2-SI): categorized as “hypointense”, “intermediate”, “hyperintense” or “mixed”, in relation to the external myometrium; (7) Signal intensity of the solid components on high b-value DWI (DWI-SI): categorized as “hypointense”, “intermediate”, “hyperintense” or “mixed”, in relation to urine; (8) Apparent diffusion coefficient (ADC) value: continuous variable, manually measured on ADC maps (avoiding tumor necrosis and cystic areas), three measurements were obtained and averaged. Discrepancies were resolved by a consensus, or a third radiologist (Cheng MY, with 16 years of experience) would serve as an arbitrator.

### Image segmentation

We accessed DICOM format images of axial FS-T2WI and DWI for each case on the PACS, and the tumor segmentation process was performed on these two sequences using ITK-SNAP (version 3.8.0). Volumes of interest (VOIs) were manually delineated along the outermost boundary in a slice-by-slice approach by a radiologist (Tan SF), and verified by the other radiologist (Cheng MY). Disagreements regarding VOI were resolved by consensus discussions, both radiologists were blinded to the pathological findings.

To ensure the reproducibility and stability of the extracted radiomics features, 30 samples were randomly selected 2 months later. One radiologist (Tan SF) then re-segmented the VOIs to evaluate the intra-observer reliability, and another radiologist (Zhang LJ) delineated the VOI according to the same procedure to assess the inter-observer consistency. The intraclass correlation coefficient (ICC) was calculated for each feature.

### Feature extraction and selection

Prior to the extraction of radiomics features, all images were preprocessed. Resampling to a voxel size of 1mm * 1mm * 1mm was performed to reduce the discrepancies related to various scanning parameters. All feature extractions and selections were performed by two radiologists who were blinded to the final histopathological diagnosis (benign vs. malignant). Then a total of 1046 radiomic features were derived from FS-T2WI and DWI sequences, respectively. These features could be categorized into four distinct classes: first-order statistical features (n=18, 1.72%), shape features (n=14, 1.34%), texture features (n=68, 6.50%), and higher-order features (n=946, 90.44%).

To minimize the influence of features with varying dimensions, Z-score normalization was performed for all extracted features, and the features were normalized to the normal distribution by mean and variance scaling. Then the primary data were randomly divided into training cohort and validation cohort with the ratio of 7:3. To reduce overfitting or selection bias in the radiomics model, a series of feature selection processes were carried out in the training cohort. First, we calculated the ICC for each radiomic feature, features with ICC<0.8 were removed from the final dataset. Second, we computed the variance of the features and remove features with outlier ratios higher than 20% based on the variance. Subsequently, univariate feature selection was implemented using the F-test combined with the SelectKBest algorithm. Features demonstrating statistically significant discriminative power (*p*-value < 0.05) were retained for subsequent multivariate modeling. This approach synergized statistical rigor with algorithmic efficiency, ensuring optimal retention of informative features.

### Model construction and validation

Univariate analysis and multivariate logistic regression analysis were successively performed based on the primary cohort to identify independent predictors for clinical and conventional MRI model. The variables with *p* values < 0.05 in the univariate logistics regression analysis were considered to have significant differences between malignant and benign SCSTs, and were included in multivariate logistics regression analysis. Based on the selected clinical characteristics and MRI parameters, logistic regression classifier was used to establish clinical model and conventional MRI model, and then combined to establish the traditional model.

In addition, based on the optimal radiomics features selected in the above procedures, extreme gradient boosting (XGBoost) was used to develop the radiomics models. The XGBoost algorithm is a scalable tree boosting system which is used widely to achieve state-of-the-art results on many machine learning challenges (13), compared to other ML methods, it was chosen for its resistance to overfitting in datasets with imbalanced feature/outcome ratios and its availability of hyperparameters (14). In addition, to address class imbalance, we set the XGBoost scale_pos_weight parameter to the ratio of negative to positive samples in the training cohort, thereby giving higher weight to the minority class. Hyperparameter tuning was performed using a grid search with 5-fold cross-validation. The search space included maximum tree depth (max_depth), learning rate (learning_rate), number of estimators (n_estimators), subsampling ratio (subsample), and column sampling ratio (colsample_bytree). The optimal combination (based on highest cross-validated AUC) was: max_depth = 4, learning_rate = 0.05, n_estimators = 200, subsample = 0.8, colsample_bytree = 0.8, gamma = 0.1, min_child_weight = 1.

The radiomics models included two models based on individual MRI sequences (FS-T2 model and DWI model) as well as a dual-sequence combined model (T2&DWI model). The calculating result of the radiomics model was named “Rad-score”. The Rad-score was integrated with the independent clinical and conventional MRI predictors to build a late fusion LR model, called the combined model, which was visualized using a nomogram.

**Figure 2:**
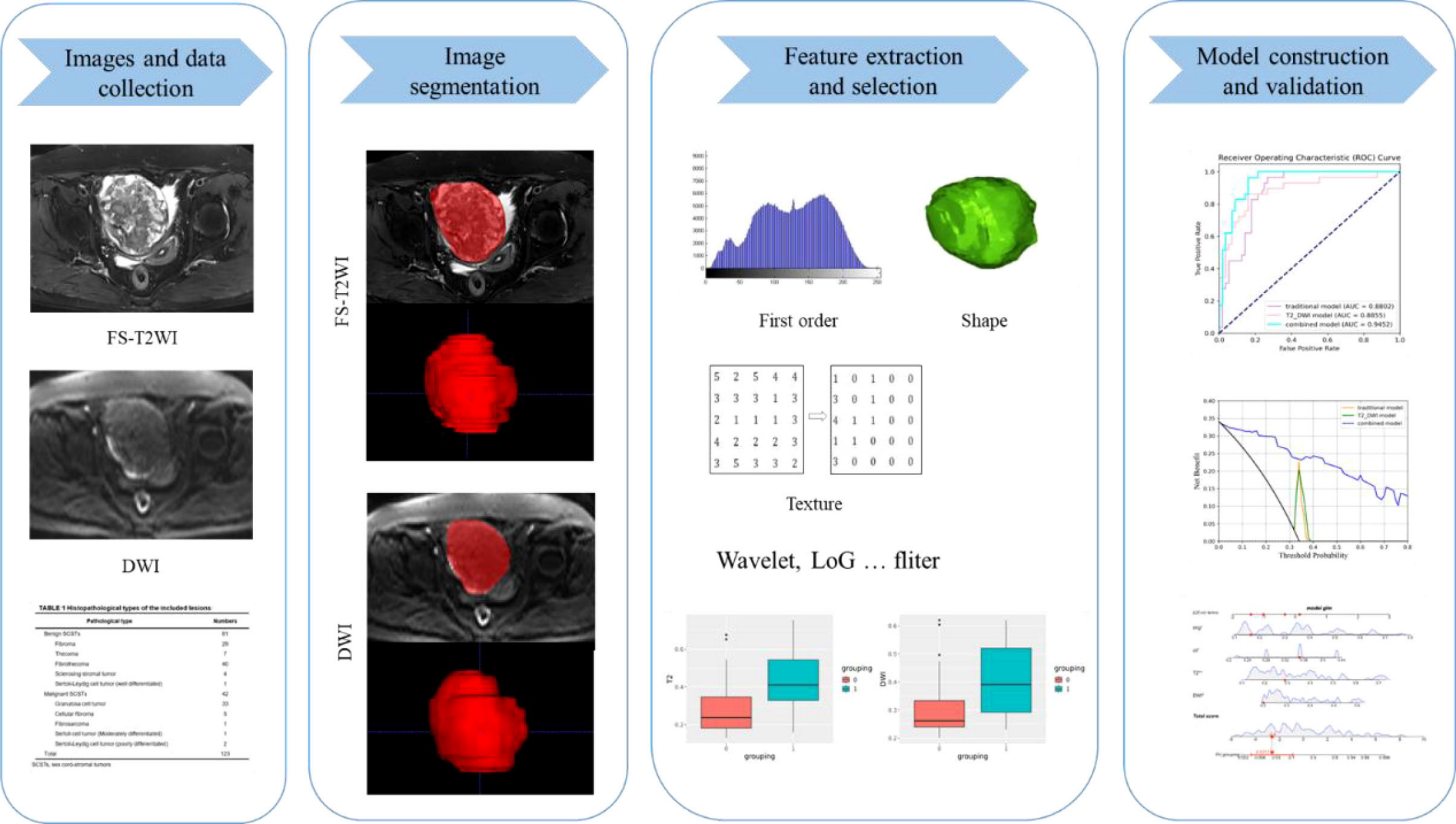
The technical workflow.

All the models above were constructed from the training cohort and tested using the validation cohort. For both training and validation sets, the diagnostic performance of all models was evaluated using receiver operating characteristic (ROC) curves, and the area under the curve (AUC), accuracy, sensitivity, specificity were calculated. Furthermore, decision curve analysis (DCA) was conducted to evaluate the clinical practicality of each model by quantifying the net benefits at various threshold probabilities. The technical workflow is illustrated in **Figure 2**.

### Statistical analysis

Statistical analyses of demographic and conventional MRI characteristics were conducted using SPSS version (v25.0, IBM Corp). Continuous variables with normal distribution were described as mean ± standard deviation, while non-normally distributed continuous variables were expressed as median and interquartile range (P25–P75). while Categorical variables were reported as frequencies and percentages (%). Group comparisons employed Chi-square tests (or Fisher’s exact test for sparse data) and independent t-tests (or Mann-Whitney U tests for nonparametric distributions). The DeLong test was used to compare the performance of different models. Two-tailed *p*-value <0.05 was considered statistically significant.

Radiomics feature extraction was performed using the PyRadiomics package (version 3.0.1), following the Image Biomarker Standardization Initiative (IBSI) guidelines. In Python, the following libraries were used: scikit-learn (version 1.0.2) for feature selection and model evaluation, XGboost (version 1.5.0) for radiomics model construction, pandas (version 1.3.5) and numpy (version 1.21.6) for data processing, scipy (version 1.7.3) for statistical analysis, and matplotlib (version 3.5.3) and seaborn (version 0.11.2) for visualization. Feature selection was implemented using scikit-learn (version 1.0.2), including variance thresholding and univariate feature selection (SelectKBest with F-test). All experiments were conducted in a fixed computational environment, and random seeds were set to ensure reproducibility.

## Results

### Patient sample

According to the inclusion and exclusion criteria, we finally enrolled 113 patients with 123 lesions as the primary cohort, out of which six patients (5.3%) had bilateral or multiple ovarian lesions. Of these, 42 lesions (34.1%) were histopathologically confirmed to be malignant, and 81 lesions (65.9%) were confirmed to be benign. Fibrothecomas constituted the majority of benign lesions (49.4%, 40/81), followed by fibromas (35.8%, 29/81), in contrast, granulosa cell tumors dominated the malignant cohort (78.6%, 33/42). The histopathological details of included lesions are presented in **Table 1**.

**Table 1:** Histopathological types of the included lesions.

Pathological type	Numbers
Benign SCSTs	81
Fibroma	29
Thecoma	7
Fibrothecoma	40
Sclerosing stromal tumor	4
Sertoli-Leydig cell tumor (well differentiated)	1
Malignant SCSTs	42
Granulosa cell tumor	33
Cellular fibroma	5
Fibrosarcoma	1
Sertoli cell tumor (Moderately differentiated)	1
Sertoli-Leydig cell tumor (poorly differentiated)	2
Total	123

SCSTs, sex cord-stromal tumors.

### Traditional analysis

We conducted a descriptive statistical analysis on the clinical characteristics and conventional MRI parameters of the two groups, the results are displayed in **Table 2** and **Table 3**. As shown, there were significant differences in menstrual status, clinical symptoms, serum CA125, pelvic effusion, tumor shape, peripheral cystic areas, lesion types, T2-SI, DWI-SI and ADC value between the benign and malignant groups.

**Table 2:** Comparison of clinical characteristics of the two groups.

Variable	Benign group (n = 81)	Malignant group (n = 42)	χ^2^/Z	*P* value
Age (years) ^a^	53(32,62)	44(39,51)	-1.444	0.149
Menstrual status ^b^			7.395	0.007*
Premenopausal	37(45.7%)	30(71.4%)		
Postmenopausal	44(54.3%)	12(28.6%)		
Clinical symptoms ^b^			10.610	0.019^*^
Asymptomatic	45(55.6%)	15(35.7%)		
Menstrual disorders	14(17.3%)	18(42.9%)		
Frequent micturition	2(2.5%)	1(2.4%)		
Abdominal pain	16(19.8%)	8(19.0%)		
Palpable lump	4(4.9%)	0(0%)		
CA125 ^b^			6.128	0.032^*^
Normal	59(73.8%)	38(92.7%)		
Mildly abnormal	16(20.0%)	3(7.3%)		
Significantly abnormal	5(6.3%)	0(0%)		
Hormone level ^b^			2.356	0.273
Normal	58(74.4%)	26(61.9%)		
Mildly abnormal	18(23.1%)	15(35.7%)		
Significantly abnormal	2(2.6%)	1(2.4%)		
Pelvic effusion ^b^			5.983	0.050*
None	33(40.7%)	26(61.9%)		
Small	32(39.5%)	13(31.0%)		
Large	16(19.8%)	3(7.1%)		

SCST, sex cord-stromal tumors; CA125, Carbohydrate antigen 125; ^a^ Data are presented as median and interquartile range (P25, P75), *P* values calculated by the Mann-Whitney U test. ^b^ Data are presented as n (%), *P* values calculated by Chi-square tests or Fisher exact test. *P values < 0.05 were considered statistically significant.

**Table 3:** Comparison of conventional MRI parameters of the two groups.

Variable	Benign SCSTs (n = 81)	Malignant SCSTs (n = 42)	χ^2^/Z	*P* value
Shape ^b^			4.354	0.037*
Regular	53(65.4%)	35(83.3%)		
Irregular	28(34.6%)	7(16.7%)		
Margin ^b^			2.236	0.135
Sharp	76(93.8%)	36(85.7%)		
Indistinct	5(6.2%)	6(14.3%)		
MD (mm) ^a^	48.70(31.10,70.40)	50.90(38.58,71.10)	-0.931	0.352
Lesion types ^b^			32.315	<0.001^*^
Predominantly solid	71(87.7%)	18(42.9%)		
Cystic-solid	9(11.1%)	11(26.2%)		
Predominantly cystic	1(1.2%)	13(31.0%)		
Peripheral cystic areas ^b^			22.852	<0.001^*^
Absent	56(69.1%)	10(23.8%)		
Present	25(30.9%)	32(76.2%)		
T2-SI ^b^			34.502	<0.001^*^
Hypointensity	26(32.1%)	1(2.4%)		
Intermediate	25(30.9%)	10(23.8%)		
Hyperintensity	16(19.8%)	29(69.0%)		
Mixed	14(17.3%)	2(4.8%)		
DWI-SI ^b^			39.574	<0.001^*^
Hypointensity	27(33.3%)	1(2.4%)		
Intermediate	22(27.2%)	2(4.8%)		
Hyperintensity	25(30.9%)	37(88.1%)		
Mixed	7(8.6%)	2(4.8%)		
ADC (×10^-3^mm^2^/s) ^a^	1.18(1.00,1.35)	0.88(0.77,0.99)	-4.822	<0.001^*^

SCST, sex cord-stromal tumors; MD, maximum diameter; T2-SI, signal intensity of the solid components on T2WI; T2WI, DWI-SI, signal intensity of the solid components on high b-value DWI; ADC, apparent diffusion coefficient; ^a^ Data are presented as median and interquartile range (P25, P75), *P* values calculated by the Mann-Whitney U test. ^b^ Data are presented as n (%), *P* values calculated by Chi-square tests or Fisher exact test. * *P* values < 0.05 were considered statistically significant.

The result of univariate regression analysis and multivariable logistic regression analysis (**Table 4**) showed that menstrual status, pelvic effusion, lesion types, DWI-SI and ADC value were independent predictors for malignant SCSTs. Then, the clinical model and conventional MRI model were established based on these independent variables.

**Table 4:** Multivariate logistic regression analysis of the variables.

Variable	Univariate analysis	Multivariable analysis
	*P* value	*P* value
Age (years)	0.095	
Menstrual status	0.008*	0.003*
Clinical symptoms	0.883	
CA125	0.020*	0.053
Hormone level	0.178	
Pelvic effusion	0.017*	0.039*
Shape	0.041*	0.207
Margin	0.145	
MD (mm)	0.897	
Lesion types	<0.001*	0.005*
Peripheral cystic areas	<0.001*	0.724
T2-SI	0.005*	0.389
DWI-SI	<0.001*	0.038*
ADC (×10^-3^mm^2^/s)	<0.001*	<0.001*

*P values < 0.05 were considered statistically significant.

### Feature extraction and selection

After ICC filtering (excluding features with ICC<0.8) and outlier removing (discarding features with outlier proportions exceeding 20%), the features were reduced to 903 for FS-T2WI sequence and 432 for DWI sequence, reflecting greater instability in DWI-derived radiomic profiles. Then, univariate F-test (α=0.05) was conducted to obtain the predictive features for constructing the final radiomics models. Specifically, 18 features were retained for FS-T2WI sequence, including 14 higher-order features (77.78% of total), 3 texture features (16.67%) and a single first-order feature (5.56%); The hierarchical categorization of features retained for DWI sequence also revealed a predominance of higher-order features (93.62%), followed by texture features (6.38%). The optimum features and their coefficients are shown in supplementary materials (**Supplementary Table 2**).

### Model construction and validation

**Figure 3:**
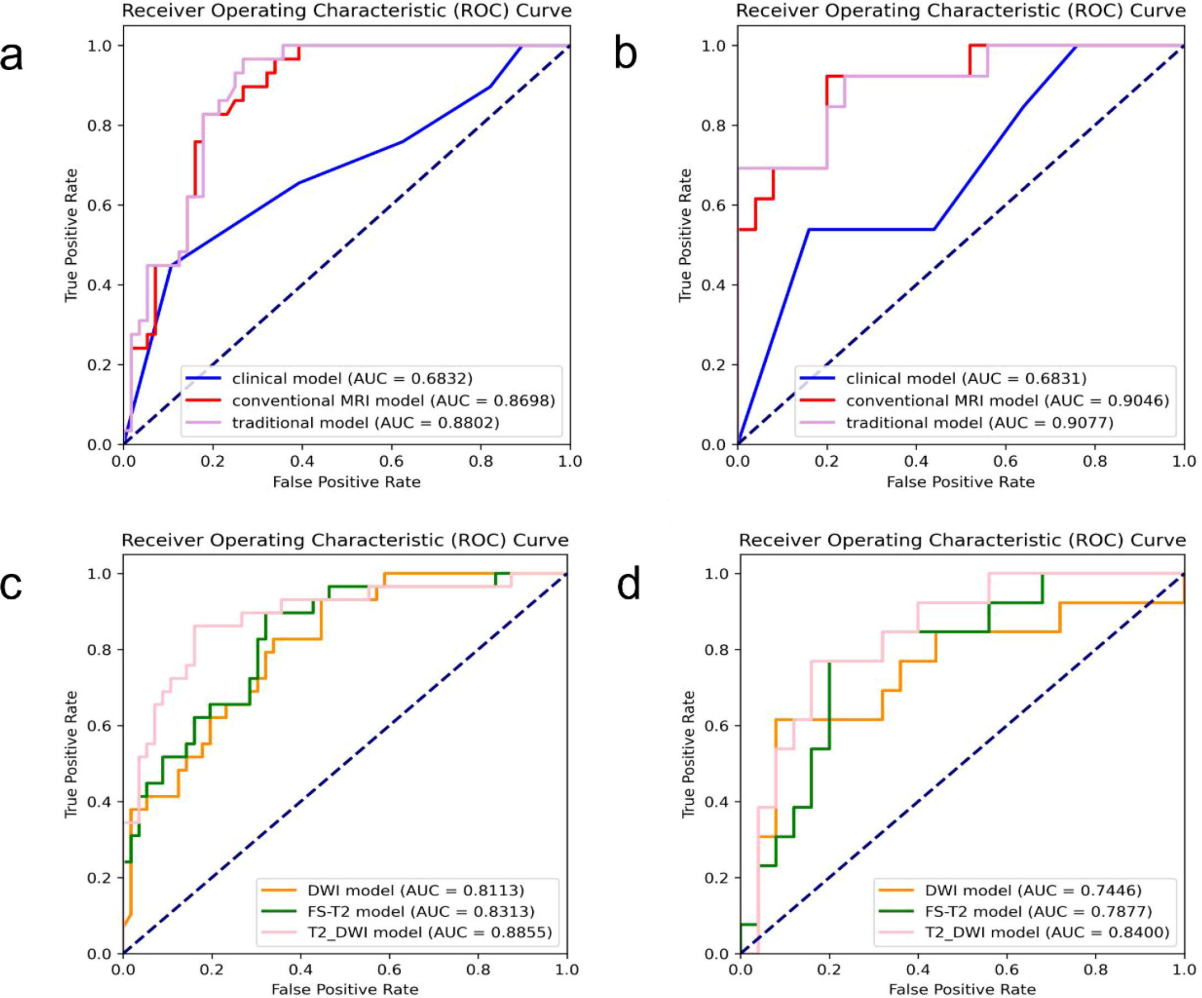
Comparative receiver operating characteristic (ROC) curves of predictive models. **(A)** Training cohort performance of the clinical model, conventional MRI model and traditional model. **(B)** Validation cohort performance of the clinical model, conventional MRI model and traditional model. **(C)** Training cohort performance of the DWI model, FS-T2 model and T2&DWI model. **(D)** Validation cohort performance of the DWI model, FS-T2 model and T2&DWI model.

In terms of traditional methods, three models were developed: The clinical model was built using menstrual status and pelvic effusion; The conventional MRI model included lesion types, DWI-SI and ADC value; The traditional model was defined as a combined model that integrated both the clinical features and the conventional MRI model. The AUCs of these three models were 0.683 (*95% CI*: 0.559-0.804), 0.870 (*95% CI*: 0.783-0.937), 0.880 (*95% CI*: 0.807-0.946) in the training cohort, respectively, and 0.683 (*95% CI*: 0.486-0.857), 0.905 (*95% CI*: 0.789-0.987), 0.908 (*95% CI*: 0.797-0.989) in the validation cohort, respectively (as shown in **Figures 3A, B**; **Table 5**). The conventional MRI model demonstrated strong predictive performance in both the training and validation cohorts. By integrating the conventional MRI model with the clinical model to establish a traditional model, the predictive performance was further improved. However, DeLong’s test revealed no statistically significant intergroup differences (*p* = 0.175 and 0.745 in the training and validation cohorts, respectively).

**Table 5:** Predictive performances of the models in the training and validation cohorts.

Model name	Training cohort				Validation cohort			
	AUC (95%CI)	ACC	SEN	SPE	AUC (95%CI)	ACC	SEN	SPE
Clinical model	0.683 (0.559-0.804)	0.506	0.759	0.375	0.683 (0.486-0.857)	0.526	0.846	0.360
Conventional MRI model	0.870 (0.783-0.937)	0.737	0.923	0.640	0.905 (0.789-0.987)	0.765	0.897	0.696
Traditional model	0.880 (0.807-0.946)	0.812	0.931	0.750	0.908 (0.797-0.989)	0.790	0.923	0.720
FS-T2 model	0.831 (0.749-0.910)	0.694	0.655	0.714	0.788 (0.608-0.925)	0.790	0.769	0.800
DWI model	0.811 (0.714-0.892)	0.729	0.621	0.786	0.745 (0.558-0.922)	0.711	0.615	0.760
T2&DWI model	0.886 (0.802-0.955)	0.835	0.862	0.821	0.840 (0.684-0.963)	0.790	0.692	0.840
Combined model	0.945 (0.892-0.985)	0.847	0.828	0.857	0.914 (0.798-0.988)	0.816	0.846	0.800

AUC, area under curve; ACC, accuracy; SEN, sensitivity; SPE, speciﬁcity.

Feature extraction from dual-sequence MRI (FS-T2WI and DWI) facilitated the construction of three models, namely FS-T2 model, DWI model, and T2&DWI model. We employed box plots (**Supplementary Figure 1**) to visualize the distribution of prediction scores generated by the two MRI sequences. The AUCs of these three models were 0.831 (*95% CI*: 0.749-0.910), 0.811 (*95% CI*: 0.714-0.892), 0.886 (*95% CI*: 0.802-0.955) in the training cohort, respectively, and 0.788 (*95% CI*: 0.608-0.925), 0.745 (*95% CI*: 0.558-0.922), 0.840 (*95% CI*: 0.684-0.963) in the validation cohort, respectively (as shown in **Figures 3C, D**; **Table 5**). Among them, the radiomics models based on a single sequence image showed moderate classification performance in the validation cohort, in contrast, the T2&DWI model which integrates both imaging sequences outperformed both in the training and validation cohorts. Nevertheless, DeLong’s test for ROC curve comparisons indicated a statistically significant difference solely between the T2&DWI model and the DWI model within the training cohort (*p* = 0.034), no significant inter-model differences were detected in other comparisons (*p* = 0.112-0.275).

**Figure 4:**
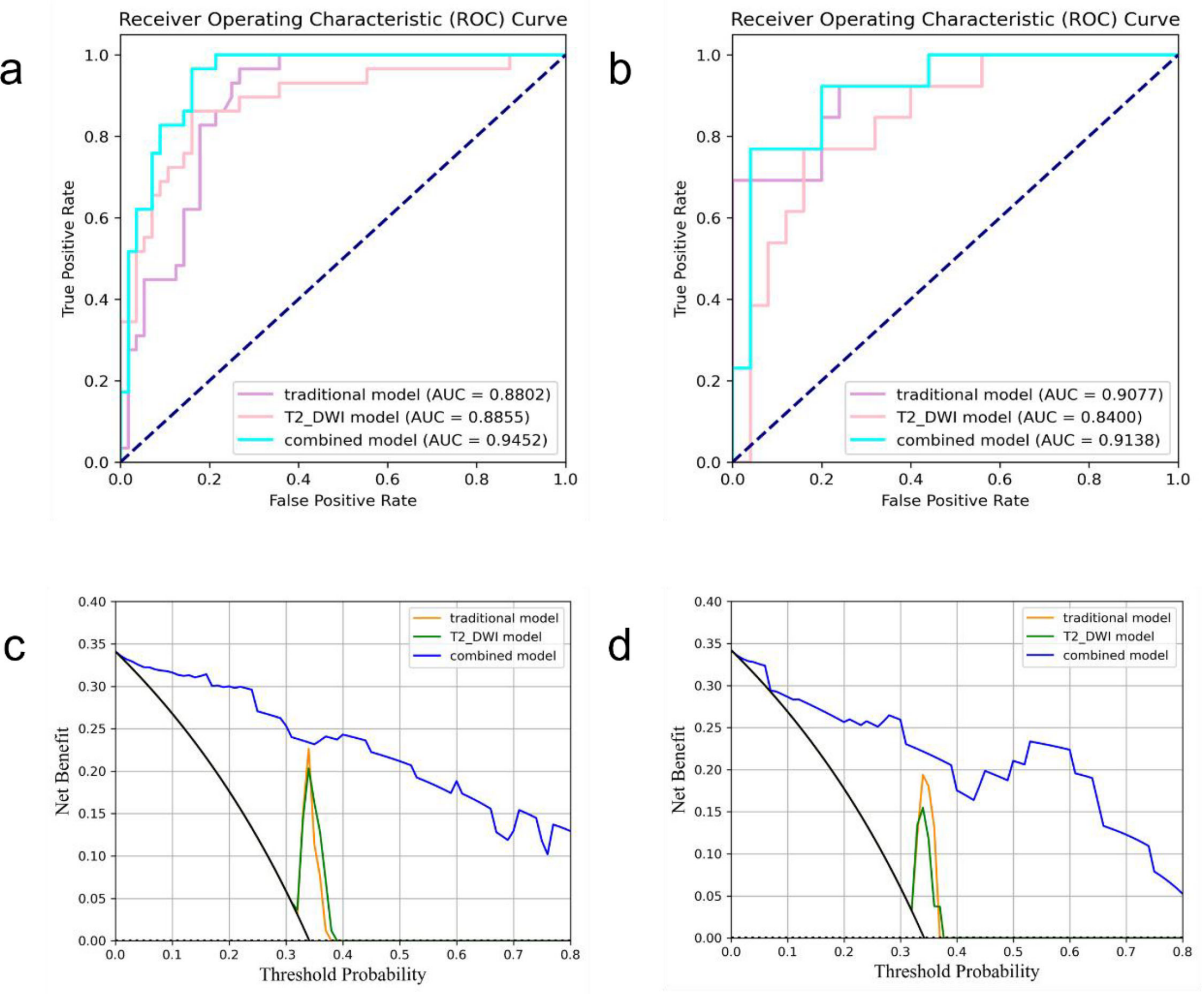
Comparative receiver operating characteristic (ROC) and Decision Curve Analysis (DCA) curves of predictive models. **(A)** Training cohort performance of the traditional model, T2&DWI model and combined model. **(B)** Validation cohort performance of the traditional model, T2&DWI model and combined model. **(C)** DCA for the traditional model, T2&DWI model and combined model in the training cohort. **(D)** DCA for the traditional model, T2&DWI model and combined model in the validation cohort.

We further integrated traditional parameters and radiomics features to develop a combined model, which achieved excellent predictive performance with AUCs of 0.945 (*95% CI*: 0.892-0.985) and 0.914 (*95% CI*: 0.798-0.988) in the training and validation cohorts, respectively (as shown in **Figures 4A, B**; **Table 5**). Subsequent comparative analysis with the traditional model and T2&DWI model revealed that, the combined model demonstrated the highest discriminative performance and accuracy among them. DeLong’s test confirmed a statistically significant improvement of the combined model over the traditional model in the training cohort (*p* = 0.018), however no significant differences were observed in other comparisons (*p* = 0.073-0.865), likely attributable to limited sample size. The decision curve analysis (DCA) revealed that for distinguishing benign from malignant SCSTs, the combined model also demonstrated superior clinical net benefit across a wide range of threshold probabilities compared to the other two models (**Figures 4C, D**).

**Figure 5:**
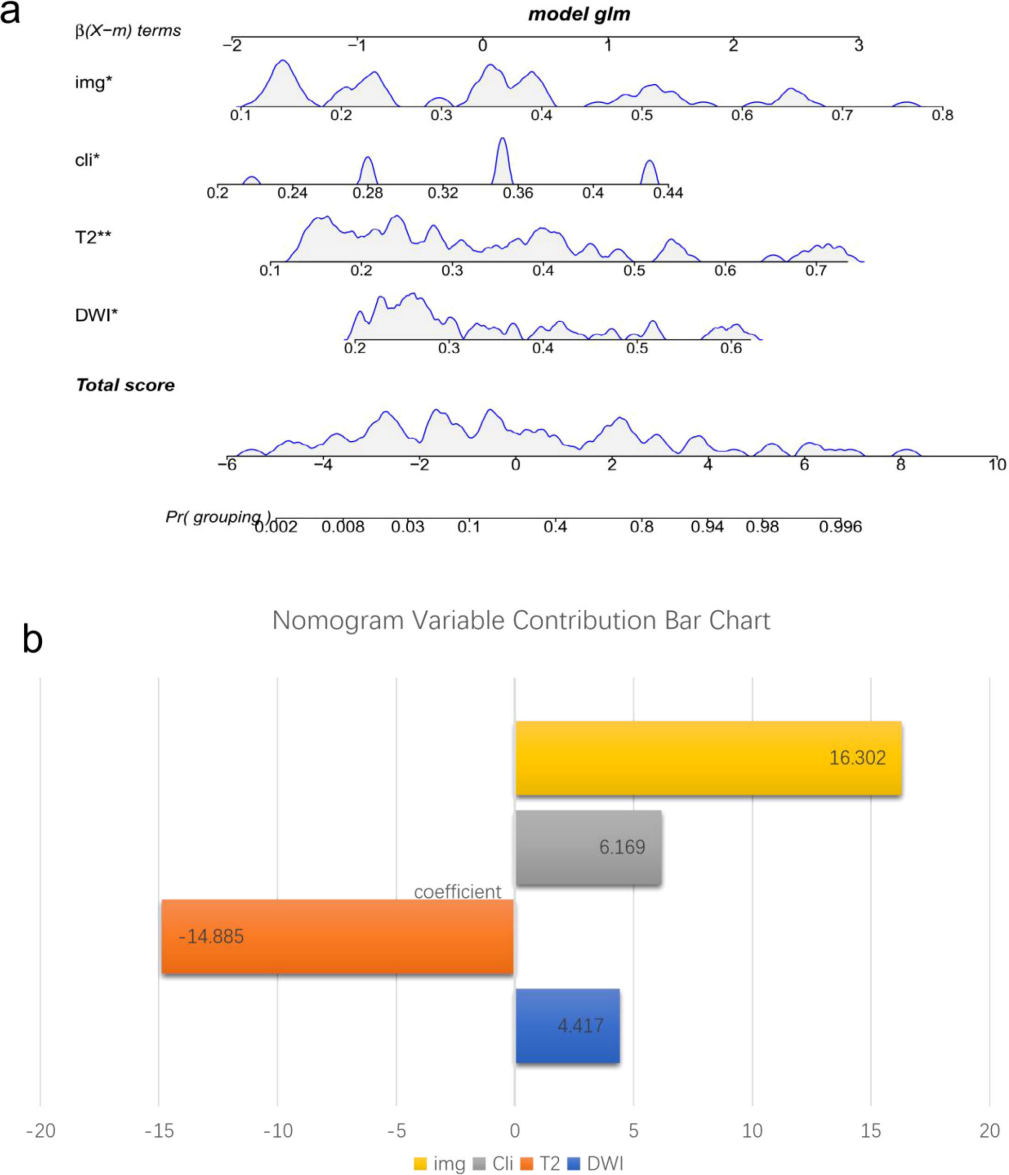
A combined model nomogram **(A)** and the nomogram variable weight decomposition **(B)** were developed. Pr(grouping) shows the predicted probability of belonging to the malignant group. Img: represents conventional MRI parameters; Cli: represents clinical features; T2: represents radiomic signatures based on FS-T2WI sequence; DWI: represents radiomic signatures based on DWI sequence.

To enhance the interpretability of the optimal model and visualize its predictive logic, we constructed a nomogram for the combined model. The nomogram graphically depicts the contributions of key predictors and quantifies individualized risk (15), as shown in **Figure 5A**. The composite risk score (Nomo-score) was calculated as: Nomo-score = (4.417×DWI) − (14.885×T2) + (6.169×cli) + (16.302×img) − 5.193. The nomogram variable weight diagram, as shown in **Figure 5B**, confirms the role of conventional MRI parameters and FS-T2WI radiomics signatures as the main predictor variables.

## Discussion

### Clinical imperative

SCSTs consist of a heterogeneous group of tumors. Malignant SCSTs, such as GCTs (as the predominant subtype), despite being classified as low-grade malignancies, exhibit non-negligible metastatic potential and late recurrence rates, which underscores the necessity for vigilant long-term follow up of these patients (2). Surgical cytoreduction remains the therapeutic mainstay for advanced GCTs patients, all visible lesions should be removed, while incomplete resection or fertility-sparing surgery (FSS) portends a worse long-term cancer-specific survival (2, 16, 17). Conversely, benign SCSTs demonstrate minimal metastatic propensity, typically allowing conservative management with fertility-preserving (18). Therefore, precise preoperative differentiation between benign and malignant SCSTs subtypes has significant implications for clinical treatment and management strategies.

### Prior studies

Prior investigations have explored imaging applications in SCSTs. Zhao et al. (2018) (9) conducted an MRI study with DWI emphasis to differentiate benign (n=29) from malignant (n=13) ovarian SCSTs. Nagawa’s study in 2022 (18) employed conventional MRI imaging combined with texture analysis to discriminate between ovarian GCTs (n=32) and thecoma−fibroma groups (n=21) in a cohort of 52 patients, developing three diagnostic approaches: MRI-based, TA-based, and combination models. In a 2023 analysis, Zhang et al. (10) compared the clinical and MRI characteristics between ovarian GCTs and other SCST subtypes involving 58 patients, establishing clinical, conventional MRI, and combined diagnostic models. Most recently, Wu et al. (2024) (19) investigated the diagnostic potential of DWI histogram analysis for differentiating ovarian GCTs from other SCST subtypes in a 27-patient cohort. As presented in the existing literature, current imaging analyses on SCSTs have some main limitations: (1) Difficulty in case collection resulting in extremely small sample sizes; (2) While SCSTs encompass diverse histological subtypes, published reports have predominantly focused on relatively common types such as GCTs and fibromas as representative; (3) Scarce application of radiomics-based methodologies, the limited existing studies have only partially incorporated basic first-order histogram or texture analysis, and these studies failed to perform essential model validation steps due to insufficient sample sizes. In contrast, although our investigation remains a small-scale study by absolute standards, it represents, to our knowledge, the largest and relatively comprehensive SCSTs imaging cohort to date. Additionally, this is the first study to implement systematic machine learning methodologies coupled with rigorous model development and validation for the classification of SCSTs.

### Clinical parameter analysis

Univariate analysis identified significant disparities in menstrual status, clinical symptoms, serum CA125, and pelvic effusion. The benign SCST cohort exhibited over half (55.6%) asymptomatic prevalence, higher than that of 35.7% in malignant cohort, the most prevalent presenting symptom was menstrual disorders, followed by abdominal pain. This was consistent with previous reports (10). SCSTs demonstrate broad age distribution at diagnosis, previous studies showed inconsistent findings regarding mean age and menstrual status. In our cohort, malignant cases demonstrated a trend toward younger presentation (± median age: 44 vs benign: ± 53 years, p=0.149), while menstrual status revealed clinically significant divergence: 71.4% of malignancies occurred in premenopausal women versus 54.3% postmenopausal predominance in benign cases (*p* < 0.05). We speculate that it may be related to the fact that symptomatic malignancies prompt earlier clinical attention, whereas benign tumors predominantly identified incidentally during physical examinations or other accidental causes, potentially delaying diagnostic confirmation. Furthermore, malignant SCSTs demonstrated a lower incidence of pelvic effusion compared to benign counterparts, particularly moderate-severe effusions, only 3/42 cases (7.1%) occurred in the malignant group, while 16/81 cases occurred in the benign group (3 cases of which were clinically diagnosed as Meigs syndrome). This finding was consistent with previous studies (10, 20, 21). Elevated serum CA125 levels were also more prevalent in benign cases, all 5 cases with significantly elevated CA125 levels were diagnosed as fibrothecomas. While the precise mechanisms remain unclear, we speculate that CA125 elevation may be associated with pelvic effusion, one possible explanation is it may be mediated by peritoneal free fluid-induced serosal surface inflammation (21, 22). However, the investigation of this association was precluded in our cohort due to limited sample size.

Following multivariate analysis, only menstrual status and pelvic effusion emerged as independent predictive factors for differentiating between benign and malignant SCSTs.

### Conventional MRI findings

In our study, malignant SCSTs demonstrated generally higher signal intensity on FS-T2WI imaging compared to benign counterparts, a finding concordant with prior reports (9, 10). The T2WI hyperintensity likely correlates with tumor degeneration and cystic changes, while different degrees of T2WI hypointensity reflect fibroblastic or fibrous tissue content (10). Regarding the role of DWI and ADC values, ESUR recommendations for MR imaging of the sonographically indeterminate adnexal mass (23) mentioned that, ADC quantification shows too much overlap to confidently allow prediction of malignancy, low DWI signal using a high b value is highly predictive of a benign lesion. We observed similar patterns: while both groups exhibited heterogeneous DWI signals, benign lesions tended to hypointensity DWI patterns versus malignant cases’ hyperintensity predominance. The slight difference was, ADC values demonstrated discriminative capacity in our cohort, with malignant lesions showing significantly lower ADC (0.88 [0.77, 0.99] ± vs 1.18 [1.00, 1.35] ± ×10^-3^ mm²/s, *p* < 0.001). Morphologically, benign SCSTs more frequently presented as solid-dominant masses (87.7% vs 42.9% malignant), with less presence of peripheral cystic areas (30.9% vs 76.2%), mirroring Nagawa’s observations (18). Multivariate analysis identified three independent discriminators: solid and cystic components, DWI-intensity, and ADC value.

### Radiomics characterization profiling

Radiomics employs high-throughput analysis of multi-dimensional quantitative features derived from medical imaging to systematically evaluate lesion characteristics and extract latent diagnostic information (11), establishing its pivotal role in oncological research. In this study, we incorporated radiomic analysis to differentiate benign from malignant SCSTs. Different imaging sequences may carry distinct information regarding tumor microarchitecture and biological behavior, our feature extraction protocol focused on two sequence: FS-T2WI, routinely employed for ovarian tumor evaluation due to its superior soft tissue contrast in delineating lesion morphology and enhanced sensitivity for cystic/necrotic components (8); and DWI, captures microstructural variations through water diffusion restriction, sensitively detecting cellular density and tumor microenvironment alterations (23). Therefore, it is reasonable to explore the discriminative ability of radiomics in SCST based on FS-T2WI and DWI sequences. Following dimensionality reduction, we identified 18 FS-T2WI derived and 47 DWI derived biomarkers, predominantly comprising high-order features, including wavelet-transformed and Laplacian of Gaussian (LoG)-filtered parameters. Radiomic features probe distinct biologic mechanisms (24): Wavelet decomposition captures tumor heterogeneity by isolating high-frequency and low-frequency components, they reflect intratumoral complexity such as macroscopic necrosis and microvascular patterns; While LoG filters enhance edge-related texture patterns that characterize tumor interface irregularity and stromal infiltration. The feature selection bias toward higher-order transformations suggests that malignancy discrimination in SCSTs relies more heavily on spatially resolved complexity metrics than on global intensity statistics. However, the biological interpretability of these complex radiomic signatures necessitates rigorous validation through histopathological correlation studies.

### Model efficacy

Through systematic model construction and comparative validation, three models demonstrated acceptable diagnostic performance: the T2&DWI model, traditional model, and combined model. The dual-sequence radiomics approach (T2&DWI model) exhibited robust discriminatory capacity, with a validation AUC of 0.840 (*95% CI*: 0.684-0.963), indicating its diagnostic utility. However, a 4.6% AUC attenuation during validation (training: 0.886 vs validation 0.840) may indicate potential overfitting to training data or limited generalizability of extracted radiomic features, underscoring the necessity for statistical consistency between the training and validation cohorts as per established guidelines (25). In contrast, the traditional model demonstrated superior stability with a marginal validation AUC improvement (Δ+2.8%, training: 0.880 vs validation 0.908), reflecting not only enhanced absolute performance but also stronger generalizability. Notably, the combined model achieved optimal discrimination in both cohorts (training AUC = 0.945, validation AUC = 0.914) and showed the best net benefit in DCA, revealing synergistic complementarity between conventional imaging biomarkers and radiomic signatures. This performance superiority substantiates the additive value of merging traditional radiological expertise with advanced computational analytics. In addition, clinical physicians are more receptive to a more intuitive and objective prediction method — nomogram. Therefore, we have developed a visual nomogram of the combined model, which can facilitate clinical translation.

### Limitations

However, our current investigation has several noteworthy limitations. First, the single-center retrospective design may constrain model generalizability, necessitating future multicenter prospective validation with larger cohorts to confirm the models’ universality and stability. Second, inherent to retrospective studies, the cohort imbalance (benign cases > malignant cases), introduces potential selection bias and reduced statistical power, potentially compromising minority class (malignant) characterization. Third, due to incomplete contrast-enhanced data availability, this study focused solely on non-contrast sequences, certain enhancement characteristics such as TIC curve patterns warrant further investigation. Fourth, the biological interpretability of radiomic features, especially higher-order statistics and wavelet-transformed features, remains limited. Unlike conventional MRI parameters, these features do not map directly to specific histopathological correlates. Therefore, the biological explanations are exploratory. Future studies incorporating radiogenomics or whole-slide histopathology correlation are needed.

## Conclusions

While both traditional model and MRI-based radiomics models demonstrate significant potential for differentiating benign and malignant SCSTs, our combined model, which synergistically integrating clinical factors, conventional MRI parameters, and dual-sequence radiomic signatures, achieves optimal predictive performance and net benefit among all models. This comprehensive approach provides a methodological advancement in SCSTs risk stratification, offering clinicians a more robust and effective tool to optimize personalized therapeutic management.

## Data Availability

The raw data supporting the conclusions of this article will be made available by the authors, without undue reservation.
