# Supplementary material for: Discrimination of benign and malignant ovarian sex cord-stromal tumors through the analysis of clinical features, MR imaging, and MR-based radiomics
Source: Front Oncol. 2026 May 25;16:1817093. doi: 10.3389/fonc.2026.1817093 (PMC13243227; doi:10.3389/fonc.2026.1817093)
Supplement: Supplementary file 1 [file Supplementaryfile1.docx]

| **TABLE S1 Representative information about the acquisition parameters** | | | | | | |
| --- | --- | --- | --- | --- | --- | --- |
| **Sequence** | **TR/TE（msec）** | **FOV**  **(cm²)** | **Matrix** | **Slice thickness (mm)** | **Flip Angle**  **(degree)** | **NEX** |
| 3.0T signa pioneer, GE Healthcare | | | | | | |
| Axial T2WI | 5518/83.2 | 30×34.1 | 288×288 | 5/6 | 110 | 2 |
| Sagittal T2WI | 5938/83.10 | 24×27.3 | 288×288 | 4/5 | 111 | 2 |
| Coronal T2WI | 4811/82.62 | 30×34.1 | 244×320 | 5/6 | 111 | 1 |
| Axial T1WI | 464/7.01 | 32×36.4 | 256×320 | 5/6 | 111 | 1 |
| axial DWI | 4000/75.2 | 34×38.6 | 128×128 | 5/6 | 90 | 6 |
|  | b=0, 1000 s/mm2, ADC map was automatically generated by post process procedure | | | | | |
| DCE | 4.79/1.80 | 36×40.9 | 260×260 | 1.4/1.4 | 15 | 0.7 |
| 3.0T Skyra, Siemens Healthcare | | | | | | |
| Axial T2WI | 3500/85 | 30×34.1 | 288×384 | 4/5 | 120 | 1 |
| Sagittal T2WI | 2520/99 | 24×27.3 | 320×320 | 5/6 | 140 | 1 |
| Coronal T2WI | 5410/85 | 30×34.1 | 288×384 | 5/6 | 120 | 1 |
| Axial T1WI | 547/19 | 24×27.1 | 288×384 | 4/5 | 120 | 1 |
| axial DWI | 5100/51 | 38×43.2 | 95×160 | 4/5 | 90 | 1 |
|  | b=0, 500, 800 s/mm2, ADC map was automatically generated by post process procedure | | | | | |
| DCE | 3.31/1.30 | 38×43.2 | 195×320 | 3/- | 9 | 1 |

T2WI, T2 weighted imaging; T1WI, T1 weighted imaging; DWI, diffusion-weighted imaging; ADC, apparent diffusion coefficient;

DCE, dynamic contrast enhanced

**TABLE S2** **Multivariate logistic regression analysis of the variables**

|  | **FS-T2WI sequence** | |  | **DWI sequence** | |
| --- | --- | --- | --- | --- | --- |
|  | **Radiomics feature name** | ***P* value** |  | **Radiomics feature name** | ***P* value** |
| 1 | wavelethhl_glrlm_longrunhighgraylevelemphasis | 0.004719 |  | logsigma30mm3d_firstorder_median | 0.001596 |
| 2 | wavelethhl_gldm_largedependencehighgraylevelemphasis | 0.005990 |  | logsigma30mm3d_firstorder_rootmeansquared | 0.001649 |
| 3 | logsigma50mm3d_firstorder_entropy | 0.007735 |  | logsigma40mm3d_firstorder_rootmeansquared | 0.002101 |
| 4 | waveletlhl_gldm_dependenceentropy | 0.007772 |  | logsigma40mm3d_firstorder_median | 0.002730 |
| 5 | logsigma50mm3d_glcm_sumentropy | 0.008767 |  | logsigma30mm3d_firstorder_mean | 0.005826 |
| 6 | logsigma40mm3d_firstorder_entropy | 0.010952 |  | logsigma40mm3d_firstorder_mean | 0.006277 |
| 7 | logsigma50mm3d_glcm_jointentropy | 0.011404 |  | original_glcm_clustershade | 0.007289 |
| 8 | logsigma40mm3d_glcm_sumentropy | 0.011609 |  | waveletlll_glcm_clustershade | 0.007475 |
| 9 | logsigma50mm3d_firstorder_maximum | 0.013760 |  | waveletlll_firstorder_skewness | 0.007521 |
| 10 | logsigma40mm3d_glcm_jointentropy | 0.015043 |  | original_firstorder_skewness | 0.007659 |
| 11 | logsigma30mm3d_glcm_jointentropy | 0.018487 |  | waveletlhl_firstorder_median | 0.015420 |
| 12 | logsigma50mm3d_gldm_dependenceentropy | 0.022013 |  | waveletlll_gldm_largedependencehighgraylevelemphasis | 0.020444 |
| 13 | original_glcm_jointentropy | 0.022243 |  | original_gldm_largedependencehighgraylevelemphasis | 0.020944 |
| 14 | wavelethlh_glszm_lowgraylevelzoneemphasis | 0.023070 |  | waveletllh_firstorder_median | 0.030273 |
| 15 | wavelethll_gldm_dependenceentropy | 0.023377 |  | wavelethll_firstorder_median | 0.033284 |
| 16 | wavelethlh_glszm_graylevelnonuniformitynormalized | 0.025742 |  | logsigma40mm3d_firstorder_skewness | 0.041750 |
| 17 | logsigma30mm3d_gldm_dependenceentropy | 0.027024 |  | wavelethll_firstorder_mean | 0.047957 |
| 18 | waveletlll_glszm_sizezonenonuniformitynormalized | 0.028032 |  |  |  |
| 19 | waveletlll_glcm_jointentropy | 0.028261 |  |  |  |
| 20 | logsigma40mm3d_gldm_dependenceentropy | 0.031582 |  |  |  |
| 21 | logsigma50mm3d_glcm_id | 0.031708 |  |  |  |
| 22 | wavelethlh_gldm_lowgraylevelemphasis | 0.032034 |  |  |  |
| 23 | waveletlhl_glcm_sumentropy | 0.032101 |  |  |  |
| 24 | waveletlhh_glszm_zoneentropy | 0.032107 |  |  |  |
| 25 | logsigma50mm3d_glrlm_runlengthnonuniformitynormalized | 0.032986 |  |  |  |
| 26 | logsigma50mm3d_glcm_idm | 0.033274 |  |  |  |
| 27 | wavelethlh_glrlm_lowgraylevelrunemphasis | 0.033681 |  |  |  |
| 28 | logsigma40mm3d_glrlm_runlengthnonuniformitynormalized | 0.034071 |  |  |  |
| 29 | logsigma50mm3d_glrlm_shortrunemphasis | 0.034791 |  |  |  |
| 30 | waveletlll_glszm_smallareaemphasis | 0.035184 |  |  |  |
| 31 | logsigma40mm3d_glcm_id | 0.035236 |  |  |  |
| 32 | logsigma40mm3d_glrlm_shortrunemphasis | 0.037868 |  |  |  |
| 33 | logsigma40mm3d_glcm_idm | 0.038048 |  |  |  |
| 34 | logsigma30mm3d_glcm_id | 0.038195 |  |  |  |
| 35 | waveletlhl_firstorder_entropy | 0.038210 |  |  |  |
| 36 | logsigma30mm3d_glrlm_runlengthnonuniformitynormalized | 0.039021 |  |  |  |
| 37 | wavelethlh_glcm_idn | 0.039204 |  |  |  |
| 38 | waveletlhh_glcm_idn | 0.040481 |  |  |  |
| 39 | wavelethlh_gldm_largedependencelowgraylevelemphasis | 0.040863 |  |  |  |
| 40 | logsigma30mm3d_glcm_idm | 0.041793 |  |  |  |
| 41 | logsigma30mm3d_glrlm_shortrunemphasis | 0.042516 |  |  |  |
| 42 | waveletlhl_glrlm_graylevelnonuniformitynormalized | 0.043749 |  |  |  |
| 43 | original_glcm_inversevariance | 0.047468 |  |  |  |
| 44 | waveletllh_gldm_dependenceentropy | 0.047516 |  |  |  |
| 45 | wavelethlh_glrlm_shortrunlowgraylevelemphasis | 0.048137 |  |  |  |
| 46 | logsigma30mm3d_glrlm_graylevelnonuniformitynormalized | 0.048353 |  |  |  |
| 47 | original_glszm_smallareaemphasis | 0.049609 |  |  |  |


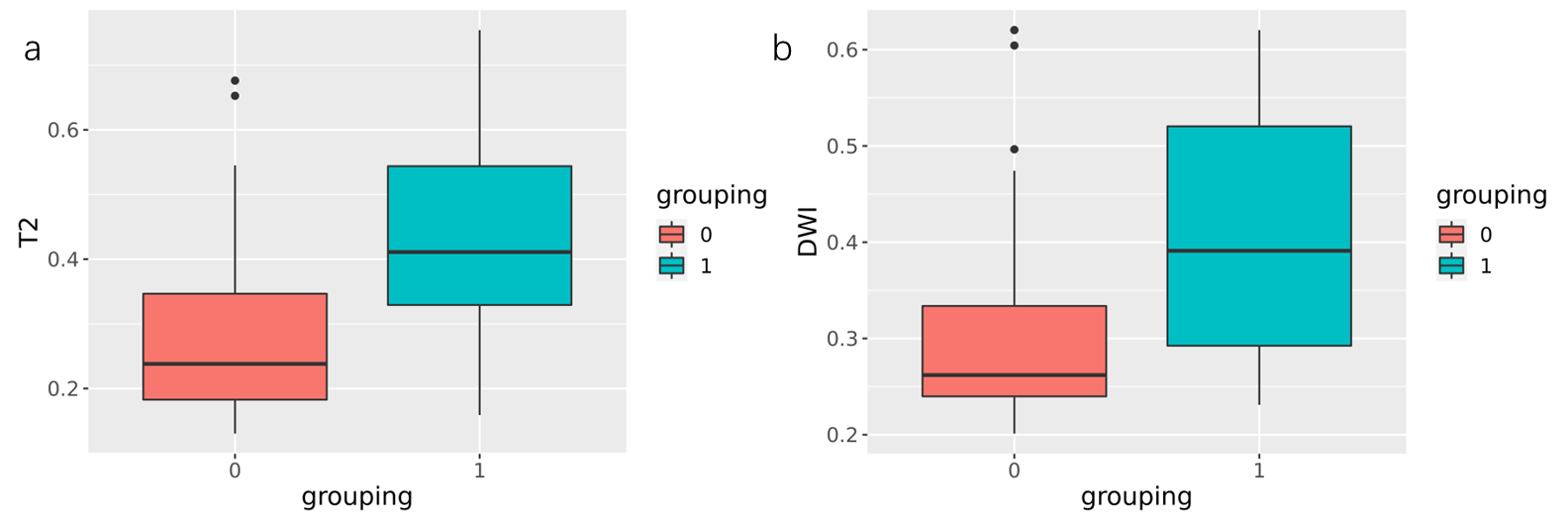


Figure S1 Box plots of the distribution of prediction scores. (a) FS-T2WI sequence, (b) DWI sequence.
